# The Association Between Internet Addiction and the Risk of Suicide Attempts in Chinese Adolescents Aged 11-17 Years: Prospective Cohort Study

**DOI:** 10.2196/52083

**Published:** 2025-01-03

**Authors:** Sihong Li, Xingyue Jin, Lintong Song, Tianqing Fan, Yanmei Shen, Jiansong Zhou

**Affiliations:** 1 Department of Psychiatry, National Clinical Research Center for Mental Disorders, and National Center for Mental Disorders The Second Xiangya Hospital of Central South University Changsha China

**Keywords:** adolescents, pathological internet use, internet addiction, suicide attempts, risk factors, cohort study

## Abstract

**Background:**

Suicide is a critical public health issue in adolescents worldwide. Internet addiction may play a role in the increased rate of suicide attempts in this population. However, few studies have explored the relationship between pathological internet use and suicide attempts among adolescents.

**Objective:**

This study aimed to conduct a prospective cohort study to examine whether higher severity of pathological internet use was associated with an increased risk of suicide attempts among Chinese adolescents.

**Methods:**

A total of 782 adolescents were recruited from a middle school from November 2020 to December 2020 and followed up for 6 months. An online self-reported questionnaire was used to collect the participants’ demographic data and assess their mental health. The Depression, Anxiety, and Stress Scale–21 items (DASS-21) was used to evaluate depression, anxiety, and stress. The Chen Internet Addiction Scale–Revised (CIAS-R) was used to assess the symptoms and severity of pathological internet use. *χ^2^* test and ANOVA were used for intergroup comparison, and logistic regression analysis was used to examine the relationship between the severity of pathological internet use and suicide attempts. We also used a restricted cubic splines model to investigate the pattern of the association.

**Results:**

The participants had an average age of 12.59 (SD 0.64) years, with the majority being of Han ethnicity (743/782, 95.01%) and more than half being male (426/782, 54.48%). Most participants had no previous history of depression (541/782, 69.18%), anxiety (415/782, 53.07%), or stress (618/782, 79.03%). The rate of newly reported suicide attempts was 4.6% (36/782). A significant positive association was observed between internet addiction and suicide attempts (odds ratio 3.88, 95% CI 1.70-8.82), which remained significant after adjusting for age, sex, ethnicity, anxiety, depression, and stress (odds ratio 2.65, 95% CI 1.07-6.55). In addition, this association exhibited a linear pattern in the restricted cubic spline regression model.

**Conclusions:**

This study suggested that internet addiction, rather than internet overuse, was associated with a higher likelihood of suicide attempts, which highlighted the importance of addressing internet addiction symptoms among Chinese adolescents for suicide prevention.

## Introduction

Suicide has emerged as a major public health concern globally recently, as it has brought significant challenges to human beings with severe consequences [1]. Over the past 5 decades, suicide remains the only contributor to rising mortality [2]. Globally, suicide ranked as the fourth leading cause of mortality among adolescents aged 15 to 19 years, as cited in a 2021 report by the World Health Organization (WHO) [3]. In the United States, it was reported that there were 2744 suicides among adolescents aged 10-19 years in 2019, positioning suicide as the second most common cause of death [3]. Therefore, reducing suicide‐related deaths has become a national priority in the United States, with more than US $22 million spent per year on suicide prevention. However, the suicide rate in the United States has not significantly declined [4]. China also faces the same challenge. Although the overall suicide rate in China has declined, the suicide rate among young people has remained high [5]. It is broadly recognized that suicide attempts may be the most significant predictor for completed suicide [6-9]. According to the data released by the National Trauma Data Bank, the mortality rate was 13% among adolescents aged younger than 20 years who attempted suicide [10]. A previous study indicated that teenagers who engaged in suicide attempts were 30 times more likely to die by suicide [11]. In addition, compared with those engaging in other forms of injury that lead to hospitalization, individuals with suicide attempts often have higher severities of injury, higher demands for health care services, and higher mortality [12]. Thus, examining the factors contributing to suicide attempts is crucial for the prevention of suicide among adolescents.

The internet has become increasingly accessible and necessary in the daily lives of adolescents. In China, internet access was available in 98.4% of elementary and middle schools in 2019 [13]. Meanwhile, in 2020, approximately 13.5% of all internet users were adolescents (aged 10-19 years) [14]. Proper internet use can help improve social skills, promote self-learning, and diversify recreation [15,16]. However, due to the highly time-consuming nature of the internet, an increasing number of adolescents have internet overuse, spending even more time using the internet than sleeping or studying [17]. In severe cases, excessive internet use may lead to clinically significant dysfunction and distress among adolescents [18,19]. This new form of behavioral addiction is listed as a tentative disorder named internet addiction. Internet addiction has also been increasing in Mainland China, with a reported prevalence of 10.4% to 26.5% among adolescents [20,21]. Earlier studies suggested a link between internet addiction and an increased incidence of suicide attempts [22,23]. A meta-analysis demonstrated that even after adjusting for demographic factors and depressive symptoms, those who experienced internet addiction continued to show a higher frequency of suicide attempts (pooled adjusted odds ratio [OR] 1.559) [23]. Some cross-sectional studies also indicated a link between internet addiction and suicidal behavior. For example, a study showed that adolescents with internet addiction had a greater likelihood of attempting suicide than those without this problem [24,25]. In addition, a 1-year follow-up study revealed that internet addiction was predictive of self-destructive behaviors among adolescents [26]. However, most previous studies are cross-sectional, which precluded the inference of causality. Furthermore, limited studies have investigated the relationship between the severity of pathological internet use and suicide attempts. Thus, in this prospective cohort study, we aimed to examine whether higher severity of pathological Internet use was associated with an increased risk of suicide attempts among Chinese adolescents [23].

## Methods

### Study Population

In this prospective cohort study, a 2-stage cluster sampling design was used to enhance the feasibility and representativeness. The sampling frame consisted of all public secondary schools across all districts in Changsha, as provided by the local education bureau. In the first stage, 1 public secondary school was randomly selected from the sampling frame. In the second stage, all students in grades 7 and 8 from the selected school were included in the study, resulting in a total sample of 1162 students (609 in grade 7 and 553 in grade 8). The selected school is comparable with most public secondary schools in the area in terms of size and demographic factors, such as gender ratio and socioeconomic background. This makes the school representative of the student population in Changsha and similar cities.

Recruitment occurred from November 2020 to December 2020, and follow-up data were collected from May 2021 to June 2021. Adolescents completed a standardized, self-administered questionnaire distributed through WeChat, one of the largest social media platforms in China. Before the commencement of the survey, we provided comprehensive training to schoolteachers on the procedures for distributing the questionnaire, offering guidance, and assisting students with difficulties. Data were collected from self-reported questionnaires administered through WeChat. All variables were collected through WeChat with the support of school personnel.

With the assistance of the school personnel, we obtained informed consent from both the adolescents and their parents. The participants accessed the survey using their parents’ mobile phones and provided electronic informed consent on the first page of the survey. Upon completion, we thoroughly checked the data to ensure the data quality. Questionnaires were considered unqualified if the completion time was excessively short, if there were evident logical inconsistencies in responses, or if critical answers were missing.

Inclusion criteria of participants for the baseline and follow-up surveys were as follows: (1) middle school students aged 11-17 years, (2) with no severe medical conditions that prevented them from completing the survey, (3) capable of comprehending and finishing the questionnaires, and (4) provided a formal agreement to participate in the research. Participants who reported suicide attempts at baseline were excluded from the follow-up study. At baseline, 1162 grade 7-8 students were recruited, among whom 78 refused to participate. Therefore, 1084 students completed the survey at baseline. For the follow-up survey, 135 individuals were excluded due to suicide attempts at baseline, and 167 were excluded for refusal to participate; thus, 782 participants were included in the follow-up survey.

### Assessment

#### Confounders

##### Sociodemographic Characteristics

Demographic variables, including sex, age, and ethnicity, were collected from the participants.

##### Depression, Anxiety, and Stress

The Depression, Anxiety, and Stress Scale–21 items (DASS-21) was used to assess participants’ levels of depression, anxiety, and stress during the previous week [27]. The assessment includes 3 subscales: Depression, Anxiety, and Stress. Each of these subscales comprises 7 items that are evaluated using a 4-point Likert scale, which ranges from 0 (not at all) to 3 (most of the time) [28]. The thresholds were set at ≥13 for depression, ≥7 for anxiety, and ≥10 for stress [27]. The DASS-21 has shown high internal consistency (Cronbach α=0.92), indicating its good cross-cultural validity [29].

### Exposures: Pathological Internet Use

Based on the conceptual framework, we used the Chen Internet Addiction Scale–Revised (CIAS-R) to assess the severity of pathological internet use [30]. The CIAS-R is a widely used instrument for evaluating pathological internet use, particularly in the Chinese context [31]. This self-reported scale includes 19 items, where each item is evaluated using a 4-point Likert scale that ranges from 1 (complete noncompliance) to 4 (complete conformity). The Chen Internet Addiction Scale (CIAS) total score varies between 0 and 76, with higher scores indicating more severe pathological internet use [32]. According to the total score, participants were further divided into 3 groups: the normal internet use group (<46), the internet overuse group (46-52), and the internet addiction group (≥53) [32]. The CIAS has proven to be a reliable and valid measure of pathological internet use among adolescents [30,33,34].

### Outcomes: Suicide Attempts

Suicide attempts refer to the act of self-injuring that a person engages in with the intention of taking their own life. In this study, suicide attempts were defined as “someone trying to hurt themselves on purpose with the intention of ending their life, but it did not succeed” to tailor to junior high school students’ comprehension levels. The question “Have you ever tried to commit suicide?” [22] was used at baseline to measure suicide attempts, and the question “Have you tried to commit suicide in the past 6 months?” was used to measure suicide attempts during the 6-month follow-up.

### Statistical Analysis

In this study, the *χ^2^* test was used to compare categorical variables across groups. Continuous data are shown using mean and SDs, and the comparison of continuous variables between groups was conducted using ANOVA. The relationship between pathological internet use and suicide attempts was examined using logistic regression analysis using 3 models. Model 1 was used to examine the unadjusted OR; Model 2 was used to examine the adjusted OR after controlling for age, sex, and ethnicity; and Model 3 was used to examine the adjusted OR after adjusting for age, sex, ethnicity, anxiety, depression, and stress. The restricted cubic splines model was used to investigate the pattern of the association between the CIAS and suicide attempts after adjusting for age, sex, ethnicity, anxiety, depression, and stress. Finally, a post hoc power analysis was performed using G*Power 3.1 (Heinrich Heine University Düsseldorf) to evaluate the statistical power of the logistic regression analysis. The modeling of restricted cubic splines was conducted with R (version 4.2.3; R Foundation for Statistical Computing), while SPSS (version 26.0; IBM) was used for all additional statistical analyses. A 2-tailed *P*<.05 indicated statistical significance.

### Ethical Considerations

This study protocol was examined and endorsed by the Ethics Committee of Central South University’s Second Xiangya Hospital (K009). With the assistance of the school personnel, we obtained informed consent from both the adolescents and their parents on the first page of the questionnaire. The personal details of the participants were kept confidential, and a distinct study identification number was allocated to every participant for data entry, management, and analysis. The participants received free psychological assessments and medical help when needed as compensation for their participation.

## Results

The analysis included 782 participants, whose average age was 12.59 (SD 0.64) years. Among these participants, more than half were male (426/782, 54.48%), and the majority were of Han ethnicity (743/782, 95.01%). Most of the participants reported no previous history of depression (541/782, 69.18%), anxiety (415/782, 53.07%), or stress (618/782, 79.03%). The rate of newly reported suicide attempts was 4.6% (36/782). Table 1 presents the comparison of demographic and clinical characteristics of participants among the normal internet use group, the internet overuse group, and the internet addiction group. The 3 groups showed significant differences in ethnicity (*P*=.03), depression (*P*<.001), anxiety (*P*<.001), stress (*P*<.001), and suicide attempts (*P*=.003). Compared with the normal internet use group, the internet overuse group and the internet addiction group were more likely to have stress, anxiety, depression, and suicide attempts after 6 months (*P*<.001 for stress, *P*<.001 for anxiety, *P*<.001 for depression, and *P*=.003 for suicide attempts).

**Table 1:** Demographic and clinical characteristics of students by pathological internet use.

Variables		NIU^a^ (n=609)	IO^b^ (n=99)	IA^c^ (n=74)	*F* test or chi-sqaure test (*df*)		*P* value	
**Age (years), mean (SD)**		12.59 (0.62)	12.60 (0.62)	12.52 (0.78)	0.38 (2779)		.68	
**Sex, n (%)**						0.18 (2)		.92
	Male	330 (54.19)	54 (54.55)	42 (56.76)				
	Female	279 (45.81)	45 (45.45)	32 (43.24)				
**Ethnicity, n (%)**						7.20 (2)		.03
	Han	580 (95.24)	97 (97.98)	66 (89.19)				
	others	29 (4.76)	2 (2.02)	8 (10.81)				
**Depression, n (%)**						52.14 (2)		<.001
	No	460 (75.53)	47 (47.47)	34 (45.95)				
	Yes	149 (24.47)	52 (52.53)	40 (54.05)				
**Anxiety, n (%)**						38.22 (2)		<.001
	No	359 (58.95)	32 (32.32)	24 (32.43)				
	Yes	250 (41.05)	67 (67.68)	50 (67.57)				
**Stress, n (%)**						54.97 (2)		<.001
	No	516 (84.73)	61 (61.62)	41 (55.41)				
	Yes	93 (15.27)	38 (38.38)	33 (44.59)				
**SA^d^ in 6 months, n (%)**						11.96 (2)		.003
	No	588 (96.55)	93 (93.94)	65 (87.84)				
	Yes	21 (3.45)	6 (6.06)	9 (12.16)				

^a^NIU: normal internet use.

^b^IO: internet overuse.

^c^IA: internet addiction.

^d^SA: suicide attempts.

**Figure 1:**
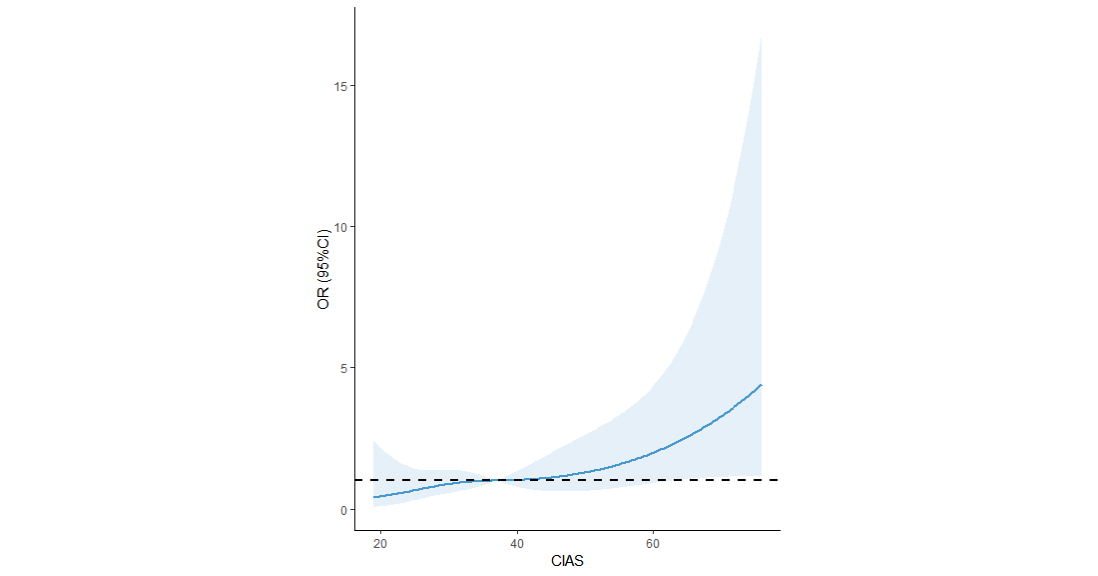
Cubic model of the association between the CIAS score and the risk of suicide attempts after adjusting for age, sex, ethnicity, anxiety, depression, and stress. CIAS: Chen Internet Addiction Scale; OR: odds ratio.

The associations between pathological internet use and suicide attempts across various models are presented in Table 2. Compared with the normal internet use group, the internet addiction group consistently showed a higher risk of suicidal attempts in Model 1 (crude OR 3.88 95% CI 1.70-8.82), Model 2 (adjusted OR 3.88, 95% CI 1.65-9.11), and Model 3 (adjusted OR 2.65, 95% CI 1.07-6.55). The restricted cubic spline regression model showed a linear association between CIAS and suicide attempts (Figure 1). Furthermore, internet overuse was also linked to an increased risk of suicidal attempts, but the association lacked statistical significance. The post hoc power analysis indicated that the statistical power of this study was 82.60% based on a sample size of 782 participants, an α level of .05, and the event probability under the null hypothesis of 3.45%.

**Table 2:** The associations between pathological internet use and suicide attempts.

Group	Suicidal attempts, n/N (%)	Model 1^a^		Model 2^b^		Model 3^c^	
		OR^d^ (95% CI)	*P* value	OR (95% CI)	*P* value	OR (95% CI)	*P* value
NIU^e^ (ref^f^)	21/609 (3.45)	1.00	—^g^	1.00	—	1.00	—
IO^h^	6/99 (6.06)	1.81 (0.71-4.59)	.21	1.90 (0.74-4.90)	.18	1.38 (0.52-3.68)	.52
IA^i^	9/74 (12.16)	3.88 (1.70-8.82)	.001	3.88 (1.65-9.11)	.002	2.65(1.07-6.55)	.04
*P* value for trend	—	—	.01	—	.01	—	.11

^a^Model 1: crude odds ratio.

^b^Model 2: odds ratio adjusted for age, sex, and ethnicity.

^c^Model 3: odds ratio adjusted for age, sex, ethnicity, anxiety, depression, and stress.

^d^OR: odds ratio.

^e^NIU: normal internet use.

^f^Ref: Reference.

^g^Not available.

^h^IO: internet overuse.

^i^IA: internet addiction.

## Discussion

### Principal Findings

To our knowledge, our research represents the initial investigation into the association between varying degrees of pathological internet use and suicide attempts among Chinese adolescents, using a prospective cohort design. Our findings suggested that the risk of suicide attempts was higher in individuals with internet addiction, even when controlling for variables such as age, sex, ethnicity, anxiety, depression, and stress. Furthermore, the restricted cubic splines model supported the linearity of the association between pathological internet use and the risk of suicide attempts.

This study found a notable association between internet addiction and suicide attempts through the 6 months of follow-up, which is in line with previous findings [35]. A study involving 8098 Chinese college students showed that those with internet addiction were at a higher risk for suicidal behaviors, with a prevalence of 21.4% [22]. Furthermore, a 1-year longitudinal study revealed that internet addiction was predictive of self-destructive behaviors, including self-harm and suicidal behaviors [26]. Furthermore, 2 meta-analyses also indicated that internet addiction was positively associated with suicidal behaviors [23,36]. Some reasons might explain this association. First, individuals with internet addiction have a greater chance of exposure to online content involving suicidal thoughts and behaviors, which may result in imitation of suicidal behaviors [37], especially for individuals with mental health problems [38]. Second, risk factors for both suicidal attempts and internet addiction, such as depression, anxiety, and low socioeconomic status, seem to overlap [39,40]. Third, alternative explanations for the link between internet addiction and suicide attempts may involve brain and cognitive dysfunctions.

For instance, internet addiction is often comorbid with some mental health disorders involving impairment of impulse control, such as attention-deficit/hyperactivity disorder and substance use disorders, suggesting the impulsive nature associated with internet addiction [41,42]. Furthermore, a magnetic resonance imaging study showed that individuals with internet addiction had impaired prefrontal lobe function, which might lead to decreased ability of impulse control or even cognitive deficits [43]. Therefore, adolescents with internet addiction may exhibit a propensity for risky decision-making, such as suicide attempts [44].

After adjusting for age, sex, ethnicity, stress, anxiety, and depression, the OR of suicidal attempts decreased from 3.88 (95% CI 1.70-8.82) to 2.64 (95% CI 1.07-6.55). Still, the link between internet addiction and suicide attempts continued to be significant. A study conducted in Korea revealed a significant positive association between internet addiction, depression, and suicidal thoughts among adolescents [45]. A study involving Chinese middle school students also pointed out that internet addiction was linked to suicide attempts among adolescents, and the association might be mediated by depression and bullying behaviors [46]. This association may also be related to the proneness of comorbidity of internet addiction and other mental health problems such as depression, decreased self-esteem, lower tolerance to frustration, and emotional distress [47,48], which also increases the risk of suicidal attempts. Some studies suggested that the connection between internet addiction and suicide attempts might be mediated by these comorbidities, which, therefore, increased the risk of suicide attempts [49].

Interestingly, this study found that internet overuse did not have a significant association with suicide attempts, indicating that suicide attempts might only be related to internet addiction, which is more severe. This aligns with an earlier study on university students, which showed that most of the mental health problems in participants with mild internet addiction were comparable with or slightly above the average. However, the rates of mental health problems sharply increased in those with moderate to severe internet addiction [50]. Some other studies suggested that engaging with the internet at levels below addiction may increase the feeling of being accepted and alleviate feelings of loneliness and shame instead of leading to severe consequences [51].

### Limitations

Some limitations need to be addressed. First, this study did not collect the specific dates of suicide attempts during the follow-up. This study only used a fixed 6-month follow-up period, which may introduce potential bias to our study results. Future studies should consider adding the specific dates of suicide attempts to get a more robust conclusion. Second, although we controlled for key risk factors such as anxiety, depression, and stress, which are known to influence suicidal behaviors, other important factors, such as academic burden and parent-child relationship, were not included in our analysis. Future research needs to include a broader range of risk factors and intermediate processes (eg, suicidal ideation) to get a more comprehensive understanding of the risk factors of suicide attempts. Third, the participants’ internet addiction and clinical correlates were collected using self-report questionnaires and scales, which might have resulted in bias such as recall bias and social desirability bias. Future studies should consider using diagnostic interviews to get more accurate assessments. Fourth, the sample was recruited from 1 middle school in Hunan Province, China, and may not represent students from other schools in other areas. Future multicenter studies across various schools and regions are needed to get a more representative sample and improve the generalizability of our results. Finally, suicide attempts were assessed based on a single self-reported question instead of a standardized scale, hindering the cross-comparison with other studies using standard measurement tools [52]. For instance, Yang et al [53] developed a standardized and reliable assessment tool for suicide attempts, which may yield more accurate and consistent data across studies. Future studies should consider using more standard tools to capture the complexity of suicidal behaviors fully.

### Conclusion

This study found that adolescents experiencing internet addiction and overuse were at a higher risk for stress, anxiety, and depression. Furthermore, internet addiction was associated with an increased risk of suicide attempts among adolescents in China. Given the considerable impact of suicide on society, the results of this study underscore the importance of addressing internet addiction among Chinese adolescents as part of suicide prevention and management efforts.

## Abbreviations

CIAS: Chen Internet Addiction Scale
CIAS-R: Chen Internet Addiction Scale-Revised
DASS-21: Depression, Anxiety, and Stress Scale–21 items
OR: odds ratio
WHO: World Health Organization

## Data Availability

The datasets generated during and/or analyzed during this study are available from the corresponding author on reasonable request.
